# Nail Changes during Pregnancy: A Cross-Sectional Survey of Patients at an Academic Center

**DOI:** 10.1159/000526870

**Published:** 2022-11-01

**Authors:** Justin Matushansky, Yu Wang, Michelle J. Chang, Charlene Thomas, Steven Hockstein, Shari R. Lipner

**Affiliations:** ^a^Leon Goldstein High School for the Sciences, New York, New York, USA; ^b^Department of Dermatology, Wake Forest School of Medicine, Winston-Salem, North Carolina, USA; ^c^Drexel University College of Medicine, Philadelphia, Pennsylvania, USA; ^d^Division of Biostatistics and Epidemiology, Weill Cornell Medicine, New York, New York, USA; ^e^Department of Obstetrics and Gynecology, Weill Cornell Medicine, New York, New York, USA; ^f^Department of Dermatology, Weill Cornell Medicine, New York, New York, USA

**Keywords:** Nails, Pregnancy, Nail growth: nail plate thickness, Leukonychia, Onychocryptosis

## Abstract

**Introduction:**

Physiological changes in skin and hair are common during pregnancy. There are limited data on nail changes during pregnancy. Therefore, our study objectives were to determine prevalence and types of nail changes in pregnant women.

**Methods:**

A prospective study was conducted in the Weill Cornell Obstetrics and Gynecology waiting room, where a 32-question survey was administered to pregnant and nonpregnant patients.

**Results:**

There was a total of 167 subjects (73 pregnant, 94 nonpregnant). Nail changes were reported by 25/73 (34.2%) and 12/94 (12.8%) pregnant and nonpregnant women, respectively (*p* < 0.05). Onychocryptosis and leukonychia were more common in pregnant (12.3% and 13.7%, respectively) versus nonpregnant women (5.3% and 0%, respectively) (*p* < 0.05). The majority of patients reported no changes in nail growth, thickness, brittleness, during their pregnancies.

**Discussion/Conclusion:**

Most nail changes in pregnant and nonpregnant women are similar. Physicians should educate women that onychocryptosis and leukonychia are common and benign findings during pregnancy.

## Introduction

Physiological changes in skin and hair are common during pregnancy. There are limited data on nail changes during pregnancy. Therefore, our study objectives were to determine prevalence and types of nail changes in pregnant women.

## Materials and Methods

A prospective and observational study was conducted in the Weill Cornell Obstetrics and Gynecology waiting room, from September 2019–December 2020, following Institutional Review Board approval. After obtaining informed consent, a 32-question survey (shown in online suppl. Table 1; for all online suppl. material, see www.karger.com/doi/10.1159/000526870) was administered to pregnant and nonpregnant patients (response rate: 99%). Patients were shown examples of common nail changes. Data were collected in REDCap, a Web-based data capture platform, which is supported by the Weill Cornell Medicine CTSC. χ^2^ and Wilcoxon-rank sum tests were used to compare categorical and continuous data, respectively.

## Results

There was a total of 167 subjects (73 pregnant, 94 nonpregnant). Pregnant participants were on average slightly younger than nonpregnant participants (29 and 31.5 years, respectively, *p* < 0.001) (shown in Table 1). Nail changes were reported by 25/73 (34.2%) and 12/94 (12.8%) pregnant and nonpregnant women, respectively (*p* < 0.05). Onychocryptosis and leukonychia were more common in pregnant (12.3% and 13.7%, respectively) versus nonpregnant women (5.3% and 0%, respectively) (*p* < 0.05). Melanonychia, Beau's lines, and onychoschizia were more frequent in pregnant versus nonpregnant women, without reaching statistical significance (shown in Table 2). Multivariate analysis showed no association between age and race with the presence of nail changes during pregnancy. The majority of patients reported no changes in nail growth, thickness, brittleness, during their pregnancies (shown in Table 2).

## Discussion/Conclusion

In our study, nail changes were relatively common in pregnancy, but only onychocryptosis and leukonychia were significantly more common in pregnant versus nonpregnant individuals. In addition, nail growth rate and thickness were unchanged for most pregnant subjects. Our study clarifies and expands upon the literature on pregnancy-associated nail changes. Previous work reported both increased and decreased nail growth rates in pregnant women compared to healthy controls [1, 2]. However, in an observational study of 94 pregnant and 82 healthy nonpregnant women, pregnancy did not alter nail growth rate or morphology but did increase nail thickness [3]. In an observational study of 312 pregnant women by Erpolat et al. [4], leukonychia was the most common nail change observed in 24.4% of patients, followed by onychocryptosis (9.0%) and onychoschizia (9.0%), observed between 14 and 42 weeks of gestation. In our study, leukonychia and onychocryptosis were also the most frequent nail changes reported in pregnant women, with similar gestational ranges as Erpolat et al. [4]. The pathophysiology of leukonychia and onychocryptosis in pregnancy has not been studied. It has been hypothesized that elevated estrogen levels may increase blood flow in the nail matrix with keratohyaline granule retention (true leukonychia) [5]. Edema may increase pressure on the nail bed vasculature, resulting in apparent leukonychia and onychocryptosis [5].

Limitations include single-center and survey-based designs, subject number, and mostly whites/non-Hispanics. Clinical examination should be included in future work to corroborate patient-reported findings since self-reported nail changes may be affected by recall bias or understanding bias. Clinical examination may identify additional nail conditions not included in the survey. True versus apparent leukonychia could not be distinguished due to study design.

In conclusion, nail changes growth, thickness, and brittleness in pregnant and nonpregnant women are similar, and contrary to popular belief, nails do not grow faster during pregnancy. Physicians should educate women that onychocryptosis and leukonychia are common and benign findings during pregnancy.

## Statement of Ethics

This study protocol was reviewed and granted exemption by Weill Cornell Medicine Institutional Review Board, number 19-05020226. Completion of the study survey constituted giving consent.

## Conflict of Interest Statement

The authors have no conflicts of interest relevant to the content of the submission.

## Funding Sources

There is no funding source.

## Author Contributions

Justin Matushansky: conception/design of work, drafting work, and final approval of version to be published. Yu Wang: acquisition, analysis, and interpretation of work; drafting work; and final approval of version to be published. Michelle J. Chang: conception/design of work, acquisition of work, drafting work, and final approval of version to be published. Charlene Thomas: analysis of work, drafting work, and final approval of version to be published. Steven Hockstein: conception/design of work, drafting work, and final approval of version to be published. Shari R Lipner: conception/design of work, analysis and interpretation of work, drafting work, and final approval of version to be published.

## Data Availability Statement

The data that support the findings of this study are available from the corresponding author upon reasonable request.

## Supplementary Material

Supplementary data

## Figures and Tables

**Table 1:** Demographics of all study patients

	Pregnant women (*N* = 73)	Non-pregnant women (*N* = 94)	*p* value
Age, median (IQR)	29 [25, 33]	32 [29,45]	<0.001
Race, *N* (%)			
White/Caucasian	53 (72.6)	70 (74.5)	0.94
East Asian	7 (9.6)	10 (10.6)	
Hispanic/Latino	5 (6.8)	4 (4.3)	
Black/African American	4 (5.5)	6 (6.4)	
South Asian	4 (5.5)	4 (4.35)	

**Table 2:** Types of nail changes

	Non-pregnant women, *N* (%)	Pregnant women, *N* (%)	*p* value	Mean gestational age in weeks (range)
All nail changes	12 (12.8)	25 (34.2)	<0.05	16.8 (4–37)
Onychocryptosis	5 (5.3)	12 (12.3)	<0.05	15.7 (8–32)
Leukonychia	0 (0.0)	10 (13.7)	<0.05	14.7 (8–37)
Longitudinal melanonychia	2 (2.1)	6 (8.2)	0.08	23.2 (12–32)
Beau's lines	3 (3.2)	6 (8.2)	0.46	20.6 (8–32)
Onychoschizia	0 (0.0)	1 (1.4)	0.6	12.0 (12)
Onycholysis	3 (3.2)	1 (1.4)	0.4	8.0 (8)
Subungual hyperkeratosis	1 (1.1)	0 (0.0)	0.99	Null
No change in nail clipping during pregnancy	No response elicited	47 (64.4)		15.7 (4–37)
More frequent nail clipping during pregnancy	No response elicited	26 (35.6)		19.1 (8–37)
No change in nail thickness during pregnancy	No response elicited	52 (71.2)		15.9 (4–37)
Increased nail thickness during pregnancy	No response elicited	21 (28.8)		19.4 (4–37)
No change in nail brittleness during pregnancy	No response elicited	63 (86.3)		16.1 (4–32)
Decreased nail brittleness during pregnancy	No response elicited	10 (13.7)		21.7 (4–37)

## References

[1] Hillman RW (1960). Fingernail growth in pregnancy: relations to some common parameters of the reproductive process. Hum Biol.

[2] Hillman RW (1955). Fingernail growth in the human subject; rates and variations in 300 individuals. Hum Biol.

[3] Altan Ferhatoğlu Z, Goktay F, Yasar S, Aytekin S (2018). Morphology, growth rate, and thickness of the nail plate during the pregnancy. Int J Dermatol.

[4] Erpolat S, Eser A, Kaygusuz I, Balci H, Kosus A, Kosus N (2016). Nail alterations during pregnancy: a clinical study. Int J Dermatol.

[5] Grossman M, Scher RK (1990). Leukonychia. Review and classification. Int J Dermatol.

