# Supplementary material for: Nail Changes during Pregnancy: A Cross-Sectional Survey of Patients at an Academic Center
Source: Skin Appendage Disord. 2022 Nov 1;9(1):27–9. doi: 10.1159/000526870 (PMC9833000; doi:10.1159/000526870)
Supplement: Supplementary file 1 — Supplementary data [file sad-0009-0027-s01.docx]

**Nail and Pregnancy Survey Questions:**

**We would like to request your participation in our study, which consists of 19 questions regarding prevalence and factors associated with nail changes during pregnancy. If you are female, you are selected as a possible participant because we are assessing nail conditions in this population.**

**Participation involves you answering all survey questions to the best of your ability. Some questions may be considered sensitive and you can choose to stop at any time. Participation is entirely voluntary and participation/non-participation will have no impact on the rights or entitlements subjects would otherwise have. The risks of participation are minimal as you will remain anonymous and there will be no way to identify participants. Therefore, you will not have access to your answers to this survey at any point during the study. You will not be identified in any reports or publications resulting from this study.**

***Completion of this study survey constitutes giving my consent to Dr. Shari Lipner and her* r*esearch team to utilize the information attained from this survey to pursue this study.***

1. **Age: _____________**
2. **Race:**

- Native American or American Indian
- South Asian (Indian, Pakistani, Bengali, etc.)
- East Asian (Chinese, Japanese, Vietnamese, Filipino, etc.)
- Black or African American
- Hispanic or Latino
- Native Hawaiian or Other Pacific Islander.
- White/Caucasian
- Other: ______________________________

1. **Have you ever been pregnant? If so, how many times? _______________**
2. **Are you pregnant?**

Yes No

**If you answered NO to question 4, skip to question 10.**

1. **Weeks into pregnancy: _______________**
2. **Do you clip your nails more or less often since you became pregnant?**

- More Often
- Less Often
- No Change
- I Don’t Know

1. **Have you noticed a change in brittleness (more ridges/splitting) in your nails since you became pregnant?**

- More Brittle
- Less Brittle
- No Change
- I Don’t Know

1. **Have you noticed a changed in thickness of your nails since you became pregnant?**

- Thicker
- Thinner
- No Change
- I Don’t Know

1. **Have you noticed your nails splitting since you became pregnant?**

- More Splitting
- Less Splitting
- No Change
- I Don’t Know

1. **How often do you receive manicures or paint your nails?**

- 3 - 4 times a month
- 1 - 2 times a month
- Rarely
- Never

1. **Do you take any vitamins or supplements? Check all that apply.**

- Prenatal
- Biotin
- Iron
- Vitamin C
- Vitamin D
- Other: _____________________________________________

**Questions 12 to 18 have a part A and B, skip Part B if you are not pregnant.**

1. **A) Do you have white spots, white streaks, or white bands on your nails? Refer to photo set 1.**

Yes No

**B) If you answered YES, was this present before pregnancy? Skip if you are not pregnant.**

Yes No

1. **A) Do the ends of your nails peel? Refer to photo set 2.**

Yes No

**B) If you answered YES, was this present before pregnancy? Skip if you are not pregnant.**

Yes No

1. **A) Do you have an indentation that runs across your nail? Refer to photo set 3.**

Yes No

**B) If you answered YES, was this present before pregnancy? Skip if you are not pregnant.**

Yes No

1. **A) Does your nail separate from the nail bed? Refer to photo set 4.**

Yes No

**B) If you answered YES, was this present before pregnancy? Skip if you are not pregnant.**

Yes No

1. **A) Do you have a brown or black band that runs down the length of your nail? Refer to photo set 5.**

Yes No

**B) If you answered YES, was this present before pregnancy? Skip if you are not pregnant.**

Yes No

1. **A) Are your nails thickened with buildup underneath your nail? Refer to photo set 6.**

Yes No

**B) If you answered YES, was this present before pregnancy? Skip if you are not pregnant.**

Yes No

1. **A) Do you have an ingrown toenail? Refer to photo set 7.**

Yes No

**B) If you answered YES, was this present before pregnancy? Skip if you are not pregnant.**

Yes No

1. **Would you be willing to have photos of your nails taken?**

Yes No
